# Supplementary material for: Non‐Hermitian Topolectrical Circuit Sensor with High Sensitivity
Source: Adv Sci (Weinh). 2023 Apr 25;10(19):2301128. doi: 10.1002/advs.202301128 (PMC10323621; doi:10.1002/advs.202301128)
Supplement: Supplementary file 1 — Supporting Information [file ADVS-10-2301128-s001.pdf]

ADVANCED SCIENCE
Open Access

Supporting Information

for *Adv. Sci.*, DOI 10.1002/advs.202301128

Non-Hermitian Topolectrical Circuit Sensor with High Sensitivity

Hao Yuan, Weixuan Zhang, Zilong Zhou, Wenlong Wang, Naiqiao Pan, Yue Feng, Houjun Sun and Xiangdong Zhang**

Supplementary Information: Non-Hermitian topolectrical circuit sensor with high sensitivity

Hao Yuan, Weixuan Zhang, Zilong Zhou, Wenlong Wang, Naiqiao Pan, Yue Feng*, Houjun Sun, and Xiangdong Zhang*

Supporting Information 1. The implementation of non-reciprocal capacitors.
Supporting Information 2. Details for the derivation of circuit eigenequations and the correspondence to the non-Hermitian topological SSH model.
Supporting Information 3. The effect of disorder on non-Hermitian topolectrical circuit sensors.
Supporting Information 4. Selection of circuit elements and the stability analysis of the circuit.
Supporting Information 5. Detailed design methods and properties of the capacitors.
Supporting Information 6. Details for the derivation of the Hermitian SSH topological circuit.
Supporting Information 7. The perturbation theory of non-Hermitian topolectrical circuit.
Supporting Information 8. The effect of the Hermitian SSH lattice with even numbers of sites.
Supporting Information 9. Noise analysis of impedance spectra of non-Hermitian topolectrical circuits.

Supporting Information 1. The implementation of non-reciprocal capacitors. In this section, we clarify the implementation of the non-reciprocal capacitor. In Supporting Figure 1, we show the composition of the non-reciprocal capacitor. The rectangle (in red) enclosed by the dotted line represents the voltage follower, which consists of an ideal operational amplifier. By connecting capacitor $2C_2$ in series with the voltage follower, and the other capacitance $(C_1 - C_2)$ in parallel is added. In this case, the non-reciprocal capacitance with the value being $C_1 \pm C_2$ is achieved.

Based on the characteristic that voltage follower can block the input current while keeping the output voltage stable, we get the following equation by carrying out Kirchhoff's law on the circuit node 1 and node 2

$$I_1 = i\omega(C_1 - C_2)(V_1 - V_2)$$

$$I_2 = i\omega(C_1 - C_2)(V_2 - V_1) + 2i\omega C_2(V_2 - V_1). \quad (1)$$

The Supporting Equation (1) can be re-expressed in a matrix form as:

$$\begin{pmatrix} I_1 \\ I_2 \end{pmatrix} = \boldsymbol{J}\begin{pmatrix} V_1 \\ V_2 \end{pmatrix} = i\omega \begin{bmatrix} -(C_1 - C_2) & (C_1 - C_2) \\ (C_1 + C_2) & -(C_1 + C_2) \end{bmatrix}\begin{pmatrix} V_1 \\ V_2 \end{pmatrix}, \quad (2)$$

where $\boldsymbol{J}$ is the admittance matrix of the circuit structure. It can be seen that the conductance matrix is a non-Hermitian matrix, and two off-diagonal elements of $(C_1 \pm C_2)$ represent effective values of the connecting capacitor between two nodes.

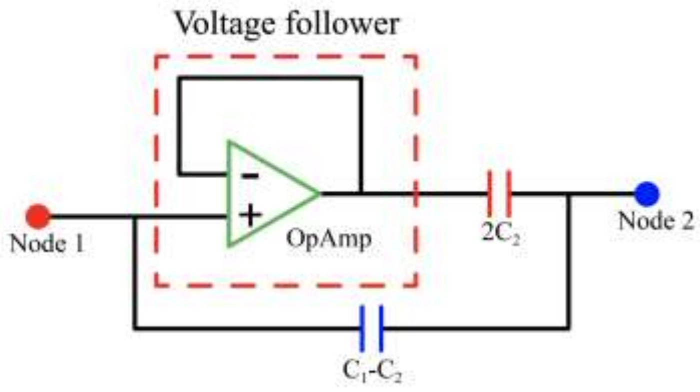

Supporting Figure 1. The model of the non-reciprocal capacitance.

Supporting Information 2. Details for the derivation of circuit eigenequations and the correspondence to the non-Hermitian topological SSH model. In this part, we give a detailed derivation of the eigenequation for the non-Hermitian topolectrical circuit and show the correspondence between the designed circuit lattice and the non-Hermitian topological SSH model. Each bulk circuit node is connected with two adjacent nodes, where one of them is connected by C_3, and the other is connected by non-reciprocal capacitor $C_1 \pm C_2$, which is $C_1 + C_2$ for odd, and $C_1 - C_2$ for even. Moreover, each circuit node is grounded by an inductor L and a grounding capacitor $2C_2$ for each odd circuit node. Carrying out Kirchhoff's law on circuit node i (odd) and j (even), we can derive the eigen-equation of the circuit as

$$\left(\frac{1}{2\omega^2 LC_2} - \frac{C_1+C_2+C_3}{2C_2}\right)V_i = -\frac{C_3}{2C_2}V_{i-1} - \frac{C_1+C_2}{2C_2}V_{i+1},$$
$$\left(\frac{1}{2\omega^2 LC_2} - \frac{C_1+C_2+C_3}{2C_2}\right)V_j = -\frac{C_3}{2C_2}V_{j+1} - \frac{C_1-C_2}{2C_2}V_{j-1}. \quad (3)$$

We provide the following identification of tight-binding parameters in terms of circuit elements as:

$$J_1 = \frac{C_3}{2C_2}, J_{2,3} = \frac{C_1 \pm C_2}{2C_2}, \varepsilon = \frac{f_0^2}{f^2} - \frac{C_1+C_2+C_3}{2C_2}, f_0 = \frac{1}{2\pi\sqrt{2LC_2}} \quad (4)$$

where J_1, $J_{2,3}$ and ε correspond to the strength of the reciprocal particle hopping, the non-reciprocal particle hopping and the eigen-energy of the non-Hermitian SSH model. In this case, Supporting Equation (3) becomes

$$\varepsilon c_i = -(J_1 c_{i+1} + J_2 c_{i-1})$$
$$\varepsilon c_j = -(J_1 c_{j-1} + J_3 c_{j+1}) \quad (5)$$

with c_i and c_j corresponding to voltages at the odd and even nodes. It is noted that Supporting Equation (5) is consistent with the eigen-equation for the non-Hermitian SSH model.

Supporting Information 3. The effect of disorder on non-Hermitian topolectrical circuit sensors. In this part, we analyze the influence of disorder on non-Hermitian topological circuits. We calculate the eigenspectra of non-Hermitian topological circuits with N=65 at different disorder strengths. The red, green, and blue hexagrams in Supporting Figure 2 correspond to cases with the disorder strength being 5%, 15%, and 30%, respectively. Topological band gaps are marked by red rectangles. Here, fifty disordered patterns are averaged. It can be seen that the size of topological band gap gets decreased by increasing the disorder strength.

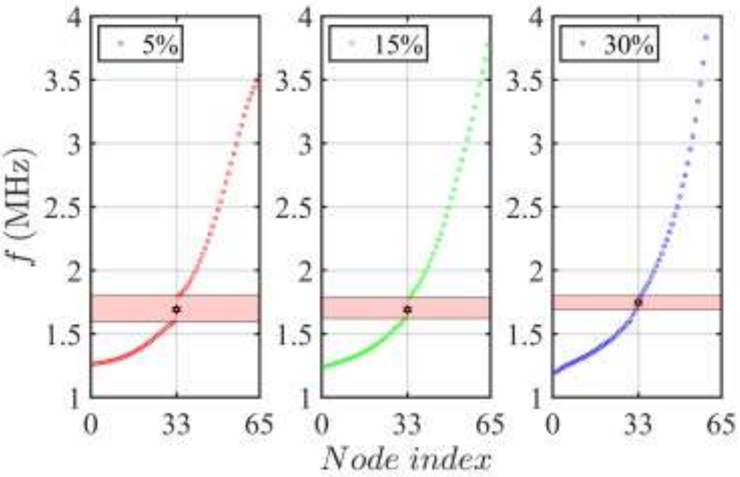

Supporting Figure 2. Under varying degrees of disorder, sorted eigenfrequencies of the finite non-Hermitian circuit with N=65. The black hexagram represents the topological zero mode.

The reduction of the topological band gap can induce the increase for the slope of frequency shifts. This effect can be clearly demonstrated in disorder-free lattices with different sizes of topological band gaps. As shown in Supporting Figure 3a, the size of the topological band gap can be easily tunned by changing the value of C_3. The frequency shift of three circuits with different sizes of topological band gaps are presented in Supporting Figure 3b, where the weak coupling capacitance C_s is 1pF. It is clearly shown that the smaller the topological band gap is, the larger for the slope of frequency shift becomes. Combining above numerical results, we can see that the disorder-induced increase for the slope of frequency shift is caused by the reduction of the associated topological band gap.

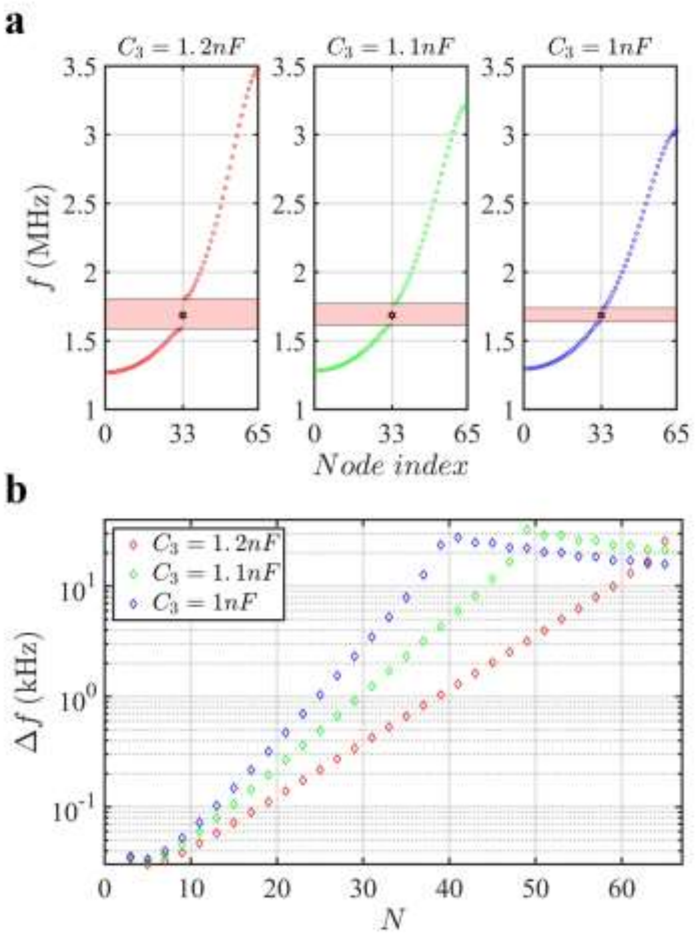

Supporting Figure 3. The effect of topological band gap size on non-Hermitian topolectrical circuit sensors. **a.** Sorted eigenfrequencies of the finite non-Hermitian circuit with N=65 for three coupling capacitors C_3. The black pentagram represents the topological zero mode. **b.** The frequency shift of topological zero mode as a function of the circuit size.

Supporting Information 4. Selection of circuit elements and the stability analysis of the circuit. In this part, we give details on the selection of circuit elements and the stability analysis of the circuit. As for the selection of inductances, we note that the loss of inductance significantly influences the value and width of the impedance peak for topological zero mode. Therefore, it is vital that the applied inductor should have a high Q value at the operational frequency. Here, we chose MWSA0603S-3R3MT of inductors, and coupling capacitors of the C0G material with 1nF and 1.2nF are used to match the operational frequency range. Additionally, unlike passive RLC circuits, there are various factors can lead to the instability of our designed circuit with active circuit elements. Hence, the selection of elements, the analysis of parasitic effects, and the layout of elements in the PCB must be considered more carefully. In this case, it is essential to analyze the stability of the circuit during the pre-experimental simulation. Here, we investigate two

different types of instabilities.

(a) The role of decoupling capacitors and their PCB layouts.

We note that the ripple generated by the rectifier used in the DC power supply can generate harmonic noises in the circuit, which will reduce the resolution and stability of the circuit. In order to smooth the ripple from the power supply, we need to connect a pair of decoupling capacitors in parallel between the power terminals of each OpAmps and the ground. In general, the decoupling capacitors with lower impedances (~1Ω) can have a good performace for filtering noises. Therefore, to make the filtering frequency range (also called as the passband) of decoupling capacitors contain the impedance peaks of our designed non-Hermitian circuit, we choose 2.2uf tantalum capacitors and 1uf multilayer ceramic capacitors as decoupling capacitors. According to the impedance spectrum of the shunt capacitor, we find that the passband of the decoupling capacitor is ranging form 0.1MHz to 700MHz (the blue rectangular), as shown in Supporting Figure 4. In this case, the decoupling capacitor can play a good role in filtering the ripple of the DC power supply in our operitional frequency region (the red rectangle). It is worth noting that the decoupling capacitor on the PCB board is placed close to the OpAmp power pins as much as possible, and the current flows through the power supply, decoupling capacitors, and OpAmp in sequence. This layout can effectively avoid distortion by external RF noise.

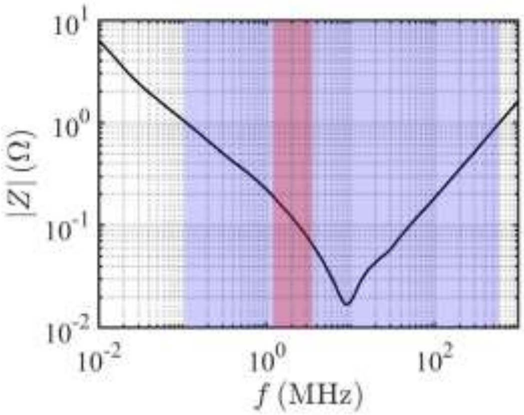

Supporting Figure 4. The frequency-dependent impadance of the decoupling capacitors used in our circuit. The blue rectangular area corresponds to the passband of the decoupling capacitor, in which the decoupling capacitor has a good filtering effect. The red rectangular area is the impedance measurement range in the experiment.

(b) The capacitive load of the OpAmp.

For the feedback circuit of the OpAmp, the phase margin is an effective quantity that can

intuitively reflect the stability and robustness of the circuit. The larger the phase margin is, the more stable the system is. However, the parasitic capacitance between the input pins of the OpAmp chip and the load package can lead to the inevitable input and output capacitances of OpAmp, which can introduce two poles into the transfer function of the feedback loop gain of the circuit. These two poles can reduce the phase margin of the circuit and decrease the stability of the system. To improve the stability of the circuit, the compensation technology should be adopted to offset the effect of the poles. The input capacitance can be compensated by connecting a feedback capacitor in parallel with the feedback resistor. The output capacitance is usually compensated by connecting an isolation resistor in series between the output pin of the OpAmp and the load. Specifically, in our designed non-Hermitian topological circuit, the isolation resistor R_2 is used to offset the effect of the output capacitor, and the negative feedback capacitor C_4 is used to offset the effect of the input capacitor, as shown in Supporting Figure 5a.

For the simple negative feedback network, we can calculate the phase margin with the help of the frequency-depedent amplitude and phase for the feedback loop gain. However, it is very difficult to calculate the feedback loop gain of our circuit because the non-reciprocal capacitor introduces a positive feedback path (marked by the red line). Here, we use a simple and intuitive method to analyze the stability by simulating the step response and overshoot of the follower module in our circuit. Specifically, the step response of a system gives information about how quickly the system responds to a sudden distortion. If the system is unstable, the step response of the system oscillates forever. In contrast, the step response of the system converges to a stable value in a stable system.

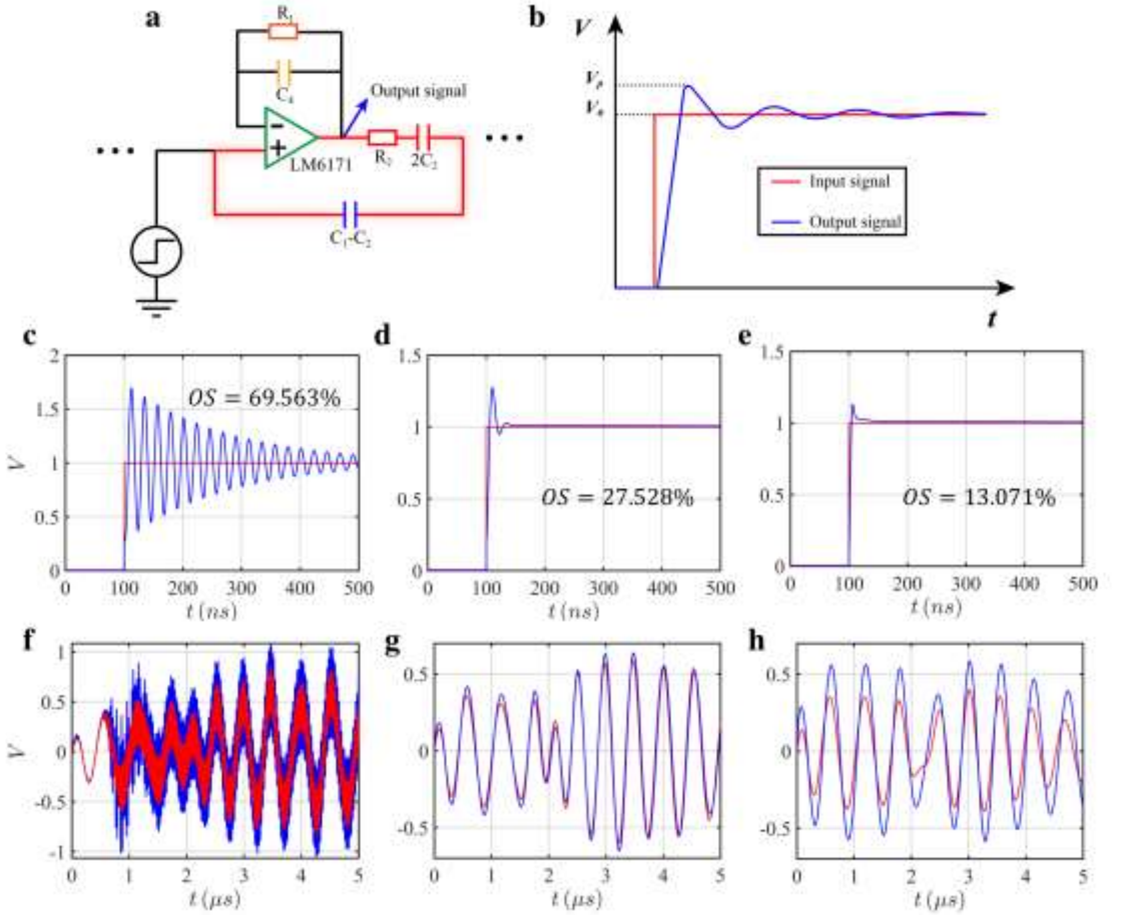

Supporting Figure 5. Simulation results of the output of voltage follower with different resistors in series. Here, we take a non-Hermitian topological circuit with 19 nodes as an example, and circuit parameters are selected in the same way as in the experiment. **a.** The scheme for the excitation and measurement of the step signal in our circuit. We input a step signal at the forward input of the OpAmp and measure it at the output of the OpAmp. **b.** Red and blue lines correspond to input and output signals in simulations. The measured results usually have the form of simulations.

c, d, e. Numerical results of step responses with the isolation resistance equaling to 0.1Ω, 5.1Ω, and 20.1Ω, respectively. **f, g, h**. Numerical results of the sine-wave (1.6861MHz) responses with the isolation resistance equaling to 0.1Ω, 5.1Ω, and 20.1Ω, respectively. As for the case of sine-wave excitation, the output signal refers to the voltage responses at the right end of resistor R_2.

In the following, we perform the stability test of the follower module in our circuit, as illustrated in Supporting Figure 5a. We inject the step signal to the non-inverting input terminal of OpAmp and measure the signal at the output terminal of the OpAmp. Red and blue lines in Supporting Figure 5b correspond to the input and output signals, where the overshoot can be expressed as $OS(\%) = 100(V_p - V_0)/V_p$. In Supporting Figure 5c-5e, we present numerical

results of the circuit with the isolation resistance equaling to 0.1Ω, 5.1Ω, and 20.1Ω, respectively. In addition, the overshoots of circuit with different isolation resistors are shown in the inside of the graph. It can be seen that the overshoot decreases significantly as the isolation resistance increases.

However, it is important to note that a too larger isolation resistor could also make the experimental phenomena become weak. In particular, when the resistor connected in series at the output of the OpAmp is too high, it can significantly reduce the voltage response of the system, and make the circuit bandwidth become narrow. In Supporting Figure 5f-5h, we calculate sine-wave responses of the circuit with different isolation resistors. It is clearly shown that the system has an obvious ringing with a small isolation resistance, where the input waveform is severely out of phase with the output waveform. When the isolation resistance is increased, there is a slight delay in the output waveform compared to the input waveform. Based on the trade-off between the stability and the response speed, a 5.1Ω resistor is chosen to be used as the isolation resistor in experiments.

Supporting Information 5. Detailed design methods and properties of the capacitors. In this part, we show the simplified capacitance models of displacement, rotation-angle, and liquid-level capacitances to determine the detailed structure parameters. Then we built a simulation model in COMSOL Multiphysics and briefly compared the experimental results with those obtained using the finite element method (FEM).

The displacement and rotation-angle capacitors can be equivalent to the parallel-plate capacitor. By ignoring the fringing effect, the capacitance value of displacement capacitance C_d and rotation-angle capacitance C_r. Specifivally, the displacement capacitance is calculated as $C_d = \frac{\varepsilon_a w d}{s}$, where ε_a is the permittivity of air, w is the width of rectangular electrodes, d is the transverse displacement, and s is the spacing of electrodes. Similarly, in the absence of fringing electric field, the capacitance value of rotation-angle capacitance C_r is calculated as $C_r = \frac{\varepsilon_a \theta r^2}{s}$, where r is the radius of semicircular electrodes, θ is the rotation angle of semicircular electrodes, and s is the spacing of electrodes. Once s is defined, C_d and C_r are proportional to d and θ, respectively.

The liquid-level capacitor is an interdigital capacitor (IDC) whose model is developed by conformal mapping. The capacitance value for two coplanar electrodes C_l is given by $C_l = \frac{2\varepsilon_l l}{\pi} ln\left[\left(1+\frac{2w}{g}\right)+\sqrt{\left(1+\frac{2w}{g}\right)^2-1}\right]$, where ε_l is the effective permittivity of sensing dielectric and l, w, and g are the length, width, and gap of electrodes, respectively. The lower limit and upper limit of ε_l are ε_a and the permittivity of measured liquid (here it is water), and ε_l is linearly related to the liquid-level t. According to the capacitance theoretical model, the designed structure parameters of three transducers are given in Table 1.

Table 1 Structure parameters of displacement, angle, and liquid level capacitances

	Displacement capacitance		Rotation-angle capacitance		Liquid-level capacitance
Length l	50mm	radius r	25mm	length l	20mm
Width w	30mm			width w	0.5mm
Spacing s	0.4mm	spacing s	0.2mm	gap g	5.0mm
Transverse displacement d	0~30mm	overapplying angle θ	0~180°	liquid level t	0~50mm

To ensure that the linearity and capacitance variation ranges of three transducers are appropriate for the proposed non-Hermitian topolectrical circuits, we built the simulation models in COMSOL Multiphysics, where the fringing effect is considered. Here, the conductive material is set to copper, silica glass is chosen as the substrate, and liquid material selects the purified water. The radius of the spherical air domain is 50cm. The FEM simulation solves Maxwell's equations for the capacitance calculation using the Electric Currents module.

The displacement capacitance in Supporting Figure 6a consists of two parallel rectangular electrodes with silica glass substrates, and the angle capacitance in Supporting Figure 6c has two parallel semicircular electrodes with silica glass substrates. Supporting Figure 6b and 6d show the experimental and simulated capacitance values in response to the displacement and angle, respectively. For the displacement capacitance, both experimental and simulated capacitance values are proportional to the relative transverse displacement d. Similarly, for the angle capacitance, both experimental and simulated capacitance values have linear relationships to the relative rotation angle θ. Note that there are discrepancies in the sensitivities of both displacement and angle capacitances between experimental and simulated data, which can be attributed to the

spacing error in the experimental install. The liquid-level capacitance in Supporting Figure 6e comprises two coplanar interpenetrating comb electrodes on the same silica glass substrate. The experimental data agree with simulation data versus the liquid-level *t* (see Supporting Figure 6f). The capacitance-level curve has a linear increase after the liquid-level is above the first electrode (5.0mm).

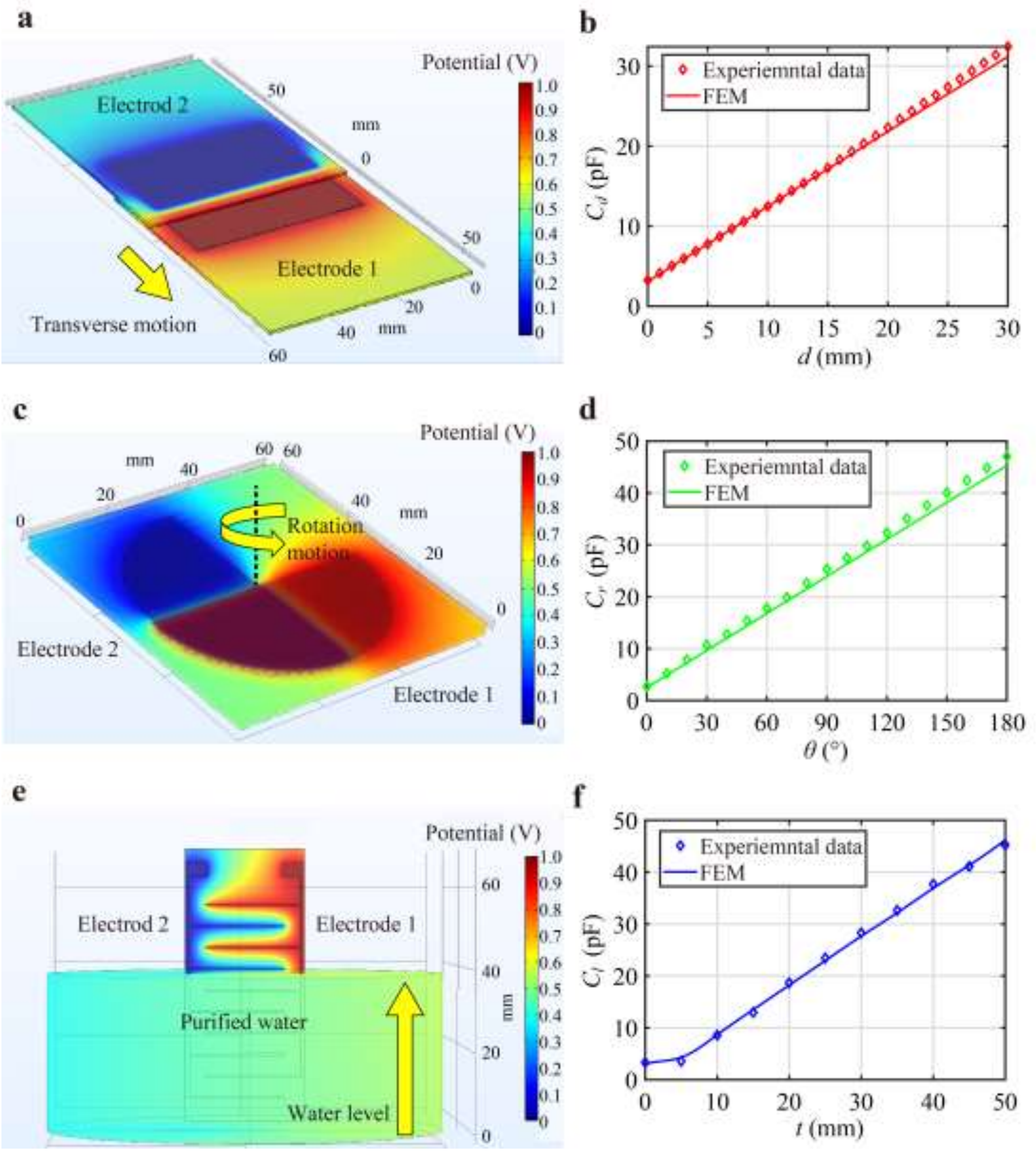

Supporting Figure 6. Simulation Models and Results of Three Capacitances. a, c, e Surface potential distributions of displacement, angle, and liquid(water)-level capacitances. The electrode 1 potential is set to 1V and electrode 2 is grounded. The spherical air domain is hidden. **b, d, f** The relationship between capacitance and three physical quantities (displacement d, angle θ, and liquid level t) in the experiment and simulation.

Supporting Information 6. Details for the derivation of the Hermitian SSH topological circuit. For comparison, in this part, we design a Hermition SSH topological circuit with an identical eigenfrequency spectrum to the non-Hermition counterpart considered in the main test.

For the non-Hermitian topolectrical circuit described in the main text, the Hamiltonian of the mapped lattice model can be written as

$$H = \sum_i^N J_2|A,i >< B,i| + J_3|B,i >< A,i| + \sum_i^{N-1} J_1|A,i >< B,i| + J_1|B,i >< A,i|. \quad (6)$$

Then, we perform a similar transformation to the Hamiltonian matrix with the similar transformation matrix S being

$$S = \begin{bmatrix} r^0 & 0 & 0 & 0 & 0 & 0 & 0 & 0 & 0 \\ 0 & r^1 & 0 & 0 & 0 & 0 & 0 & 0 & 0 \\ 0 & 0 & r^1 & 0 & 0 & 0 & 0 & 0 & 0 \\ 0 & 0 & 0 & r^2 & 0 & 0 & 0 & 0 & 0 \\ 0 & 0 & 0 & 0 & r^2 & 0 & 0 & 0 & 0 \\ 0 & 0 & 0 & 0 & 0 & \cdots & 0 & 0 & 0 \\ 0 & 0 & 0 & 0 & 0 & 0 & r^{N-1} & 0 & 0 \\ 0 & 0 & 0 & 0 & 0 & 0 & 0 & r^{N-1} & 0 \\ 0 & 0 & 0 & 0 & 0 & 0 & 0 & 0 & r^N \end{bmatrix}. \quad (7)$$

where *r* is an arbitrary value. The similarly transformed eigenequation is written as

$$H'|\varphi' >= E|\varphi' >. \quad (8)$$

where the new eigenstates satisfy $|\varphi' >= S^{-1}|\varphi >$ and the transformed Hamiltonian matrix is in the form of

$$H' = S^{-1}HS = \begin{bmatrix} 0 & rJ_2 & 0 & 0 & 0 & 0 & 0 \\ r^{-1}J_3 & 0 & J_1 & 0 & 0 & 0 & 0 \\ 0 & J_1 & 0 & rJ_2 & 0 & 0 & 0 \\ 0 & 0 & 0 & \cdots & 0 & 0 & 0 \\ 0 & 0 & 0 & r^{-1}J_3 & 0 & J_1 & 0 \\ 0 & 0 & 0 & 0 & J_1 & 0 & rJ_2 \\ 0 & 0 & 0 & 0 & 0 & r^{-1}J_3 & 0 \end{bmatrix}. \quad (9)$$

Since we wish to obtain an Hermitian by a similar transformation, we need to satisfy: $r^{-1}J_3 = rJ_2$.In this case, we have $r = \sqrt{J_3/J_2}$. In this case, the intracell (ν) and intercell (τ) coupling strengths can be expressed as

$$\nu = \sqrt{J_2 J_3} = \frac{\sqrt{C_1^2 - C_2^2}}{2C_2}, \tau = J_1 = \frac{C_3}{2C_2}. \quad (10)$$

The schematic diagram of the associated SSH circuit is shown in Supporting Figure 7. Carrying out Kirchhoff's law on circuit node *i* (odd) and *j* (even), we can derive the eigen-equation of the circuit as:

$$\left(\frac{1}{2\omega^2 LC_2} - \frac{C_x+C_y+C_3}{2C_2}\right)V_i = -\frac{C_3}{2C_2}V_{i-1} - \frac{C_x}{2C_2}V_{i+1},$$

$$\left(\frac{1}{2\omega^2 LC_2} - \frac{C_x+C_y+C_3}{2C_2}\right)V_j = -\frac{C_3}{2C_2}V_{j+1} - \frac{C_x}{2C_2}V_{j-1}. \quad (11)$$

We provide the following identification of tight-binding parameters in terms of circuit elements as:

$$\tau = \frac{C_3}{2C_2}, \nu = \frac{\sqrt{C_1^2-C_2^2}}{2C_2} = \nu, \varepsilon' = \frac{f_0^2}{f^2} - \frac{C_x+C_y+C_3}{2C_2}, f_0 = \frac{1}{2\pi\sqrt{2LC_2}} \quad (12)$$

where τ, ν correspond to the strength of the reciprocal hoppings and ε' is the eigen-energy of the Hermitian SSH model. As expected, the above circuit has an identical eigen-spectrum to that of the non-Hermitian topological circuit.

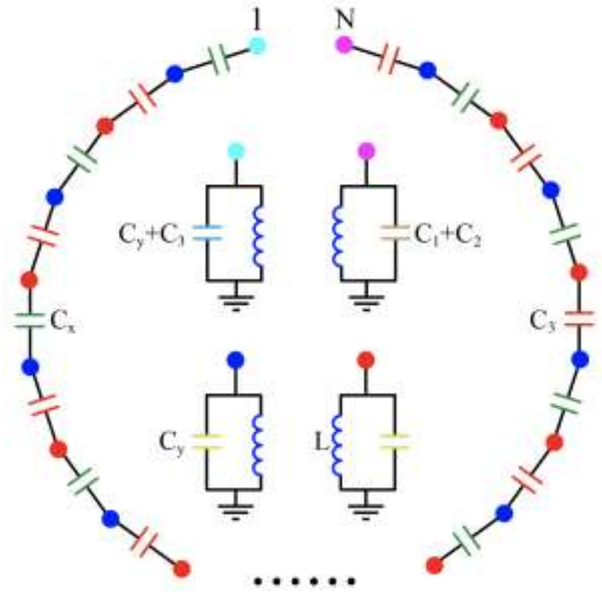

Supporting Figure 7. Schematic diagram of Hermitian topolectrical circuits.

Supporting Information 7. The perturbation theory of non-Hermitian topolectrical circuit. In this part, we give the detailed calculation of the first-order perturbation theory with boundary disturbance. The non-Hermitian SSH model we used in the main text can be described by the non-Hermitian topolectrical circuit with $2N-1$ nodes and the corresponding matrix with the form of

$$\boldsymbol{J}=\begin{bmatrix} 0 & \frac{C_1+C_2}{C_1} & 0 & 0 & 0 & \cdots & \frac{C_S}{C_1} \\ \frac{C_1-C_2}{C_1} & 0 & \frac{C_3}{C_1} & 0 & 0 & \cdots & 0 \\ 0 & \frac{C_3}{C_1} & 0 & \frac{C_1+C_2}{C_1} & 0 & \cdots & 0 \\ 0 & 0 & \frac{C_1-C_2}{C_1} & 0 & \ddots & \ddots & 0 \\ 0 & 0 & 0 & \ddots & \ddots & \frac{C_1+C_2}{C_1} & 0 \\ \vdots & \vdots & \vdots & \ddots & \frac{C_1-C_2}{C_1} & \ddots & \frac{C_3}{C_1} \\ \frac{C_S}{C_1} & 0 & 0 & \cdots & 0 & \frac{C_3}{C_1} & 0 \end{bmatrix}, \tag{13}$$

which satisfy $\boldsymbol{J}|V_{R,f}>=\varepsilon|V_{R,f}>$ and $<V_{L,f}|\boldsymbol{J}=<V_{L,f}|\varepsilon^*$, where $\varepsilon=f_0^2/f^2-(C_1+C_2+C_3)/2C_2$ with $f_0=1/\sqrt{8\pi^2LC_2}$. The C_s denotes an effective boundary perturbation, and $|V_{R,f}>$ and $<V_{L,f}|$ are right and left eigenvectors in the form of

$$|V_{R,f}>=\xi_R\begin{pmatrix} 1 \\ 0 \\ r_R \\ 0 \\ r_R^2 \\ 0 \\ r_R^3 \\ \vdots \\ 0 \\ r_R^{N-1} \end{pmatrix};<V_{L,f}|=\xi_L(1\ 0\ r_L\ 0\ r_L^2\ 0\ r_L^3\ \cdots\ 0\ r_L^{N-1}), \tag{14}$$

where $r_R=-\frac{C_1+C_2}{C_3}$, $r_L=-\frac{C_1-C_2}{C_3}$. When $C_s=0$, the frequency of topological zero mode is $f=\frac{1}{2\pi\sqrt{(C_1+C_2+C_3)L}}$. Consider that $\Delta\boldsymbol{J}=i\omega C_s(|1><2N-1|+|2N-1><1|)$, and the overlap between the left and right states is $<V_{L,f}|V_{R,f}>=\xi_R\xi_L\frac{(r_Rr_L)^N-1}{r_Rr_L-1}$. Baced on the first-order perturbation theory in C_s, we obtain

$$\Delta f\approx\frac{<V_{L,f}|\Delta\boldsymbol{J}|V_{R,f}>}{<V_{L,f}|V_{R,f}>}=C_s\frac{(r_Rr_L-1)(r_R^{N-1}+r_L^{N-1})}{(r_Rr_L)^N-1}\xrightarrow{N>>1}\propto C_se^{\eta N}. \tag{15}$$

As we can see, the frequency shift of topological zero-energy mode (Δf) is a function of the the boundary perturbation (C_s) in the case of the circuit with a fix length. The origin of the exponential-sensitive can also be proved in Supporting Equation (15).

In addition, we calculate the variation of energy shifts of topological zero-energy modes (ΔE) as a function of the strength for the boundary perturbation (Γ) with lattice length being *N*=11, *N*=15 and *N*=19, as shown in Supporting Figure 8. It can be seen that the longer the lattice is, the higher the slope appears. Such a phenomenon is consistent with the experimental results of circuit sensors with different lengths.

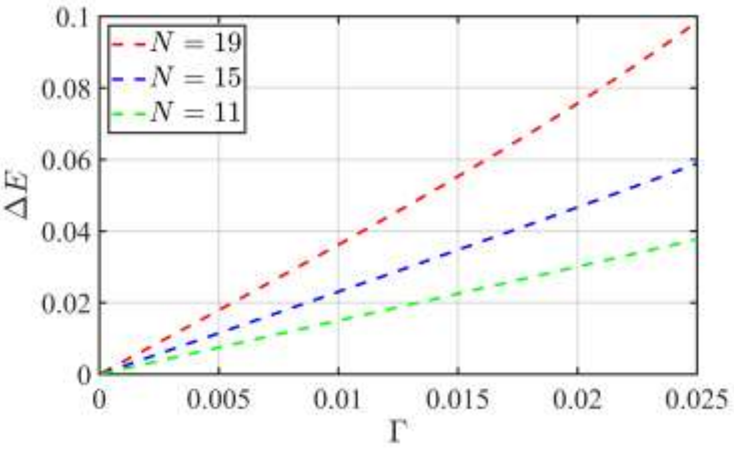

Supporting Figure 8. The variation of energy shifts of topological zero-energy modes (ΔE) as a function of the strength for the boundary perturbation (Γ) with lattice length being *N*=11, *N*=15 and *N*=19.

Supporting Information 8. The effect of the Hermitian SSH lattice with even numbers of sites. In the part, we calculate the variation of real and imaginary parts of eigenenergies for two midgap topological states as a function of the lattice length N. Supporting Figure 9a and Supporting Figure 9b correspond to cases with $N = 4n + 2$ and $N = 4(n + 1)$ (n=0, 1, ...), respectively. The red and blue dots present shifts of two different topological modes. The strength of boundary perturbation equals to 1e-3. For comparison, we also presents the related results for the lattice with odd sites ($N = 2n + 3$ with n=0, 1, ...), as shown in Supporting Figure 9c. It is clearly shown that the exponential sensitivity only exists when the imaginary part of eigenenergies for topological zero modes are zero. It can be seen that the transition points of N, where the energies change from the real value to the complex value, are different for two even-site systems with $N = 4n + 2$ and $N = 4(n + 1)$ (n=0, 1, ...).

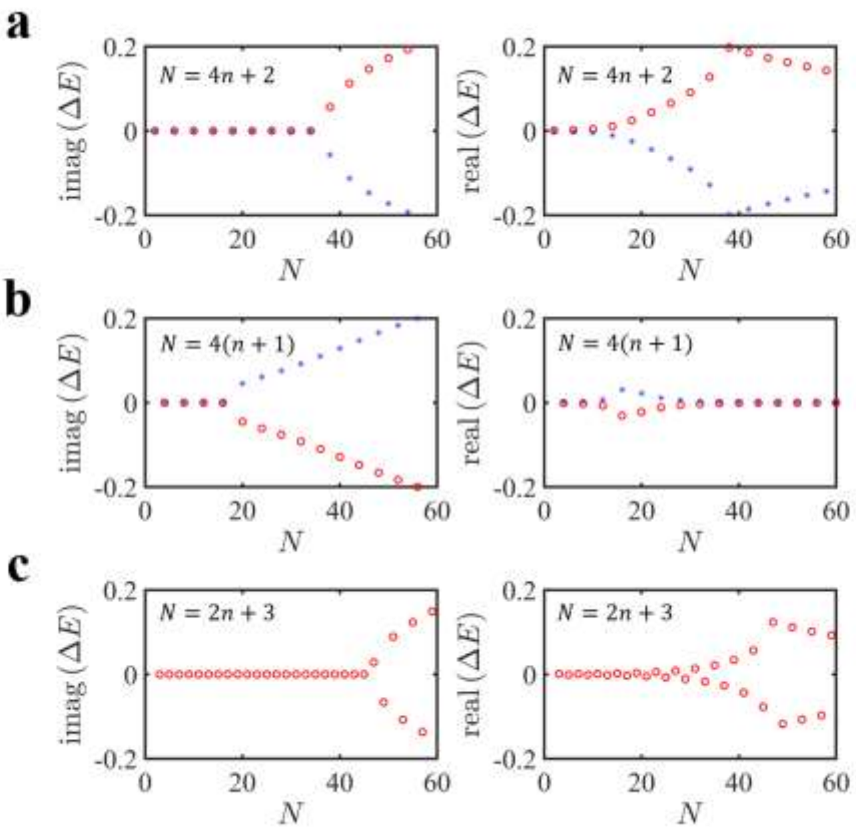

Supporting Figure 9. a, b,The variation of real and imaginary parts of eigenenergies for two midgap topological states as a function of the lattice length *with* $N = 4n + 2$ and $N = 4(n + 1)$ (n=0, 1, ...). **c.** The variation of real and imaginary parts of eigenenergies for the midgap topological state as a function of the lattice length with $N = 2n + 3$ (n=0, 1, ...).

In addition, it should be noted that there are some limitations on using non-Hermitian topological circuit sensors with even nodes. As shown in Supporting Figure 10a, eigenenergies of two topological modes in the system without boundary perturbations do not equal to zero due to the finite size effect. As shown in Supporting Figure 10b, we simulate the impedance spectrum for the system with N=30 (consider loss effect in experiments). In this case, two frequencies of impedance peaks related to two topological modes (marked by blue dashed lines) cannot be accurately identified, which can influence the precision of detected quantities.

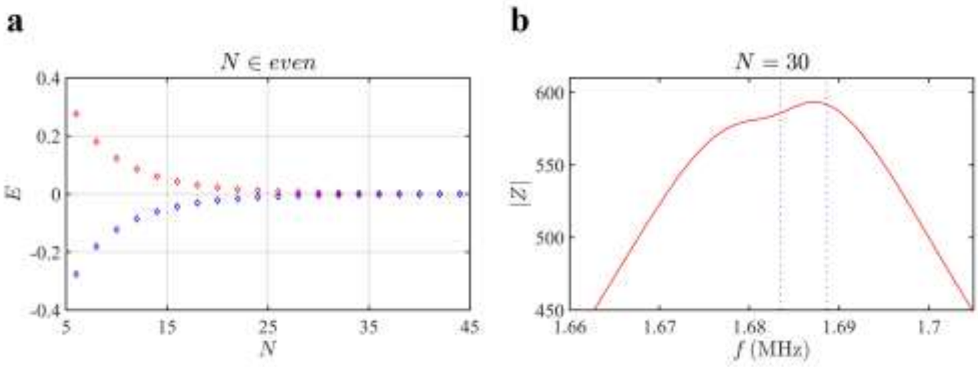

Supporting Figure 10. a. The eigenenergies of two topological modes in the system without boundary perturbations for the even-site lattice. **b.** The impedance spectrum in topological bandgap for the system with N=30 (consider loss effect in experiments).

Supporting Information 9. Noise analysis of impedance spectra of non-Hermitian topolectrical circuits. In the part, we give the noise analysis of impedance spectra of non-Hermitian topolectrical circuits. The impedance spectrum Z_{in} can be obtained using the S-parameter method. Supporting Figure 11 illustrates the scheme for the S-parameter simulation, where the resistance Z_0 (we call it as the signal-source resistor) is connected in series with the power supply, and the grounded resistance Z_0 plays the role of the impedance matching. The transfer function between two ends of the signal-source resistor can be expressed as $H(f) = V_2/V_1 = Z_{in}/(2Z_{in} + Z_0)$. In this case, the impedance Z_{in} of the circuit can be extracted from the reflection coefficient $S_{11} = \frac{-Z_0}{2Z_{in}+Z_0} = \frac{2V_2}{V_1} - 1$ with $Z_{in} = \frac{Z_0}{2} - \frac{Z_0 V_1}{2(2V_2 - V_1)}$.

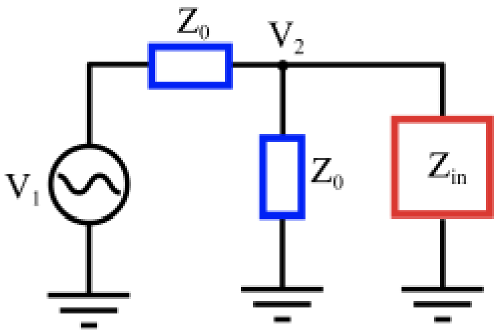

Supporting Figure 11. The scheme for the S-parameter simulation. The supply voltage V_1 is $1V$, and Z_0 takes 50Ω. The grounded resistor Z_0 plays the role of impedance matching, and Z_{in} is the impedance of the non-Hermitian topolectrical circuit.

We use the noise-simulation function in the LTspice software to solve the spectral density of voltage noises e_n for the circuit in the frequency domain, where the supply voltage V_1 in the

S-parameter simulation is $1V$ and the temperature is 27°C. The effective value of voltage noise (E_{rms}) is obtained based on the relationship $E_{rms} = \sqrt{\int e_n^2 df}$. Thus, the input impedance affected by noise can be expressed as $Z_{in} = \frac{Z_0}{2} - \frac{Z_0 V_1}{2(2(V_2+V_{noise})-V_1)}$, where the voltage noise V_{noise} is a complex Gaussian distribution satisfying a mean squared deviation of E_{rms}.

In order to suppress the influence of voltage noises on the impedance spectrum, non-Hermitian topological circuits are further optimized. In purticular, the thermal noise of feedback resistors and the inherent low-frequency noise in circuits can be suppressed by increasing the value of feedback capacitance and compensating a grounded resistance. Specifically, the feedback capacitance in the voltage follower is set as 100 times (100nF) larger than that used in the experiment, and all nodes are compensated with a 10kΩ grounding resistance. The parasitic resistance of the inductor is 100mΩ. In Supporting Figure 11, we presented the calculate impedance spectra of circuits with different values of the capacitor Cs, that connects the first and last circuit nodes, and different lengths of circuits. The red dotted line indicates the position of the zero-energy frequency of the circuit with open boundaries.

We firstly focus on the case with Cs=10 pF (the same to the experiment). Supporting Figure 12a presents the calculated impedance spectra with noises for the circuit with the length being N=13 (in red), N=17 (in green), N=21 (in blue), and N=25 (in pink), respectively. It is clearly shown that the voltage noise has a little effect on the impedance. Then, we decrease the value of Cs =1 pF, and the corresponding simulation results are shown in Supporting Figure 12b. We can see that the influence of noises on the impedance spectrum increases gradually with respect to the circuit length. It is shown that the impedance peak can still be accurately read out. While, when the value of Cs is decreased to 0.1 pF, the circuit length for realizing the maximum frequency shift of topological zero mode should be further increased. In this case, the impedance peak can not be precisely identified under noises, as shown in Supporting Figure 12c with N=41 (in red), N=49 (in green), N=57 (in blue), and N=65 (in pink). Therefore, we find that the noise effect can generate a theoretical limitation on the maximum length of non-Hermitian topolectrical circuit sensors with available performances. In this case, we note that there is an optimal length for our designed electric circuits possessing high-level sensitivity and resolvability at the same time. We also want to point out the influence of noises can be further minimized by applying our

non-Hermitian topolectrical circuit sensors at chip scale (see discussion in the main test).

Finally, it is important to note that the above calculated noise actually contains both the inherent noise of the circuit and the environmental interference noise, such as the industrial frequency noise, the RF noise, etc. Since the environmental noise can be significantly suppressed by applying the appropriate shielding devices, and the impedance analyzer can also suppress the noise with the help of lock-in amplifier, the impedance noises in experiments are much lower than the results of our theoretical simulation.

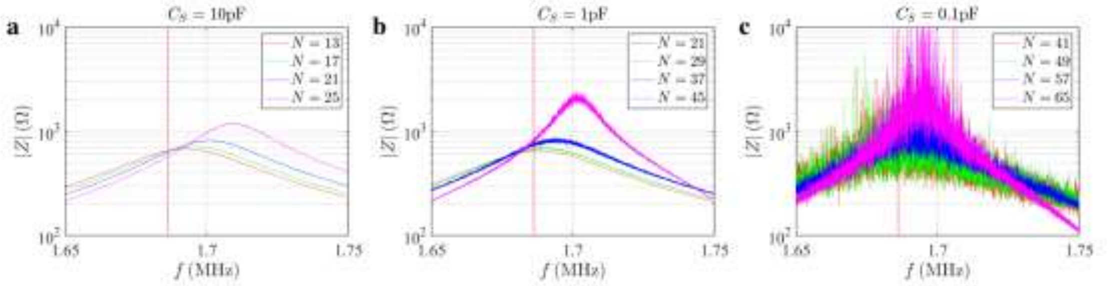

Supporting Figure 12. Simulation results of impedance spectra under the influence of noises. The parasitic resistance of the inductor is $100\mathrm{m}\Omega$. The red dotted line indicates the position of the zero-energy frequency. The feedback capacitance in the voltage follower becomes 100 times (100nF) larger than in the experiment, and all nodes compensate for a $10\mathrm{k}\Omega$ grounding resistance. The rest of the components are configured as in the experiment. **a.** The weak coupling capacitance is 10pF, and the four different chain lengths correspond to *N*=13 (red), *N*=17 (green), *N*=21 (blue), and N=25 (pink), respectively. Noise has little effect on impedance. **b.** The weak coupling capacitance is 1pF, and the four different chain lengths correspond to *N*=21 (red), *N*=29 (green), *N*=37 (blue), and *N*=45 (pink), respectively. With the increase of chain length, the influence of noise on the impedance spectrum increases gradually, and the position of the impedance peak can be read out relatively accurately. **c.** The weak coupling capacitance is 0.1pF, and the four different chain lengths correspond to *N*=41 (red), *N*=49 (green), *N*=57 (blue), and *N*=65 (Pink). With the increase in chain length, the position of the impedance peak can not be identified due to noise.
